# Supplementary material for: Accelerated maturation of branched organoids confined in collagen droplets
Source: Lab Chip. 2025 Jul 29;25(19):5043–54. doi: 10.1039/d5lc00287g (PMC12366776; doi:10.1039/d5lc00287g)
Supplement: LC-025-D5LC00287G-s001 [file LC-025-D5LC00287G-s001.pdf]

Supporting information for "Accelerated maturation of branched organoids confined in collagen droplets"

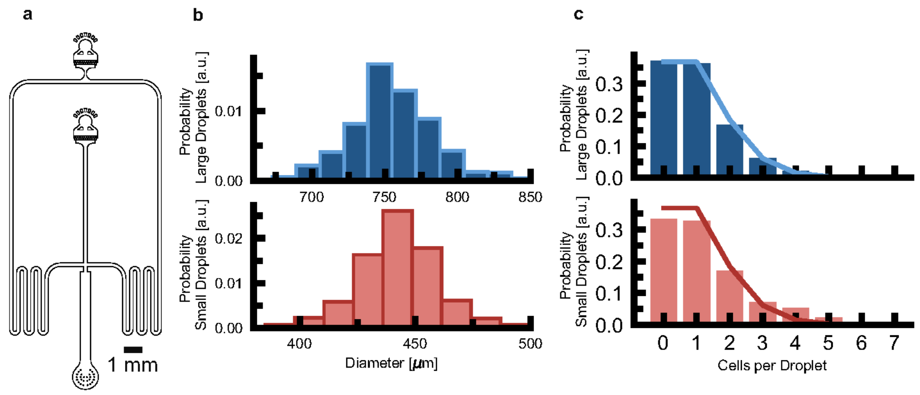

Figure S 1 Characterisation of the microfluidic setup. (a) The microfluidic chip design. (b) The different heights of the microfluidic devices allow for the generation of large and small droplets, with an average size of 750 μm and 370 μm. The histogram for the droplet size distribution is shown in the upper row for large droplets (n = 666 droplets) and in the lower row for small droplets (n = 907 droplets). (c) The incorporation frequency follows Poisson statistics. The cell incorporation frequency for large droplets is displayed in the upper row (n = 951), and the cell incorporation frequency for small droplets is shown in the lower row (n = 906). The line plot represents the theoretical Poisson distribution probabilities, assuming an average of one cell per droplet.

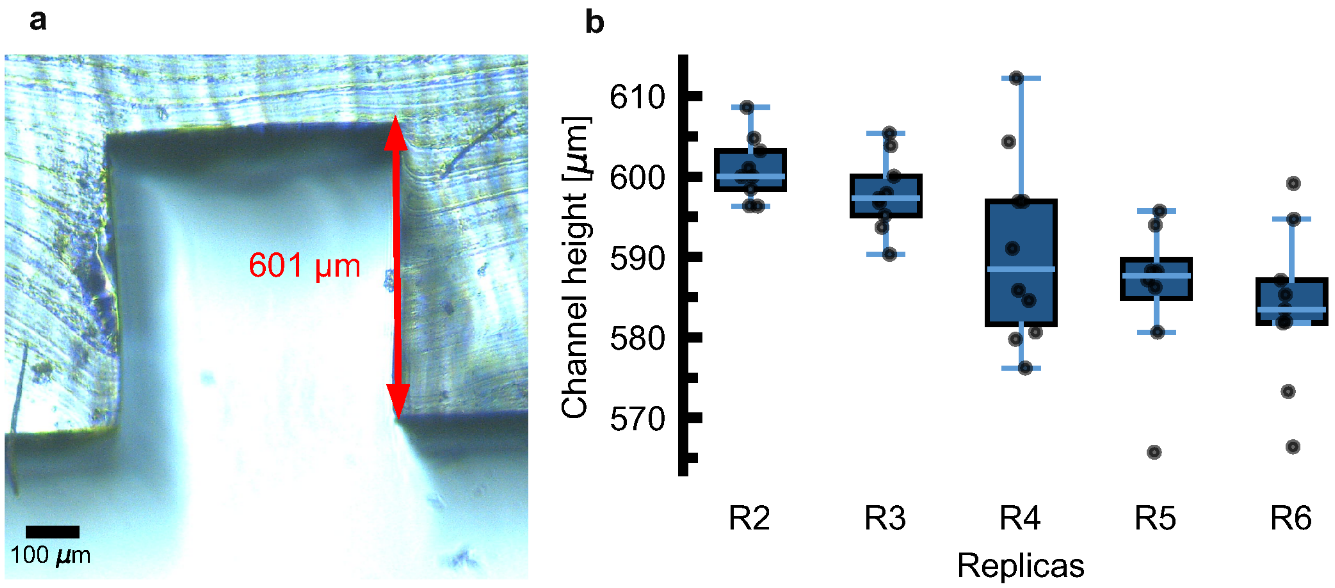

Figure S 2 Characterisation of SU8 channel height across replicas on the same master wafer. (a) The PDMS replica was cut into several sections. The height of the PDMS channels was measured at multiple positions using brightfield microscopy. (b) We quantified the channel heights for each replica on the SU8 master. Only the replicas with heights closest to the target value of 600 μm, which exhibited minimal intra-replica variation, were selected for droplet production. Data for replicas 2 to 6 are shown (n $>$ 8 distinct positions per replica). Replica 1 was excluded from analysis due to breakage during PDMS removal.

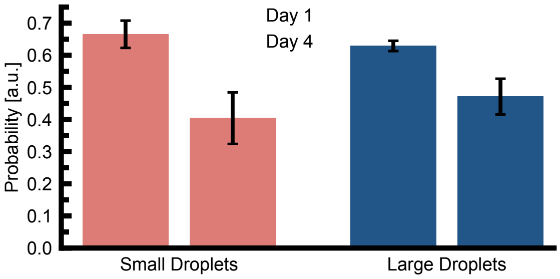

Figure S 3 Viability of droplet-grown organoids. Four days after cell seeding, 40 ± 8% of the small droplets (red) contained at least one viable organoid structure compared to 47 ± 5% of the large droplets (blue) (N = 3 independent experiments with $n_{Small} \geq 301$ and $n_{Large} \geq 300$ droplets evaluated for each experiment). The error bars show the standard deviation.

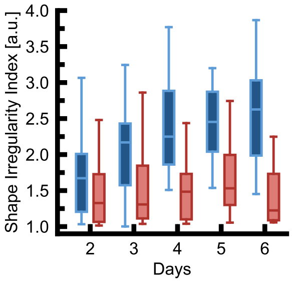

Figure S 4 Shape irregularity index of 2D-projected organoid shapes. Shape irregularity index of the 2D projected shape of the organoids grown in large droplets (blue) and in small droplets (red) over time ($n_{Large} \geq 34$ and $n_{Small} \geq 24$ for each day).

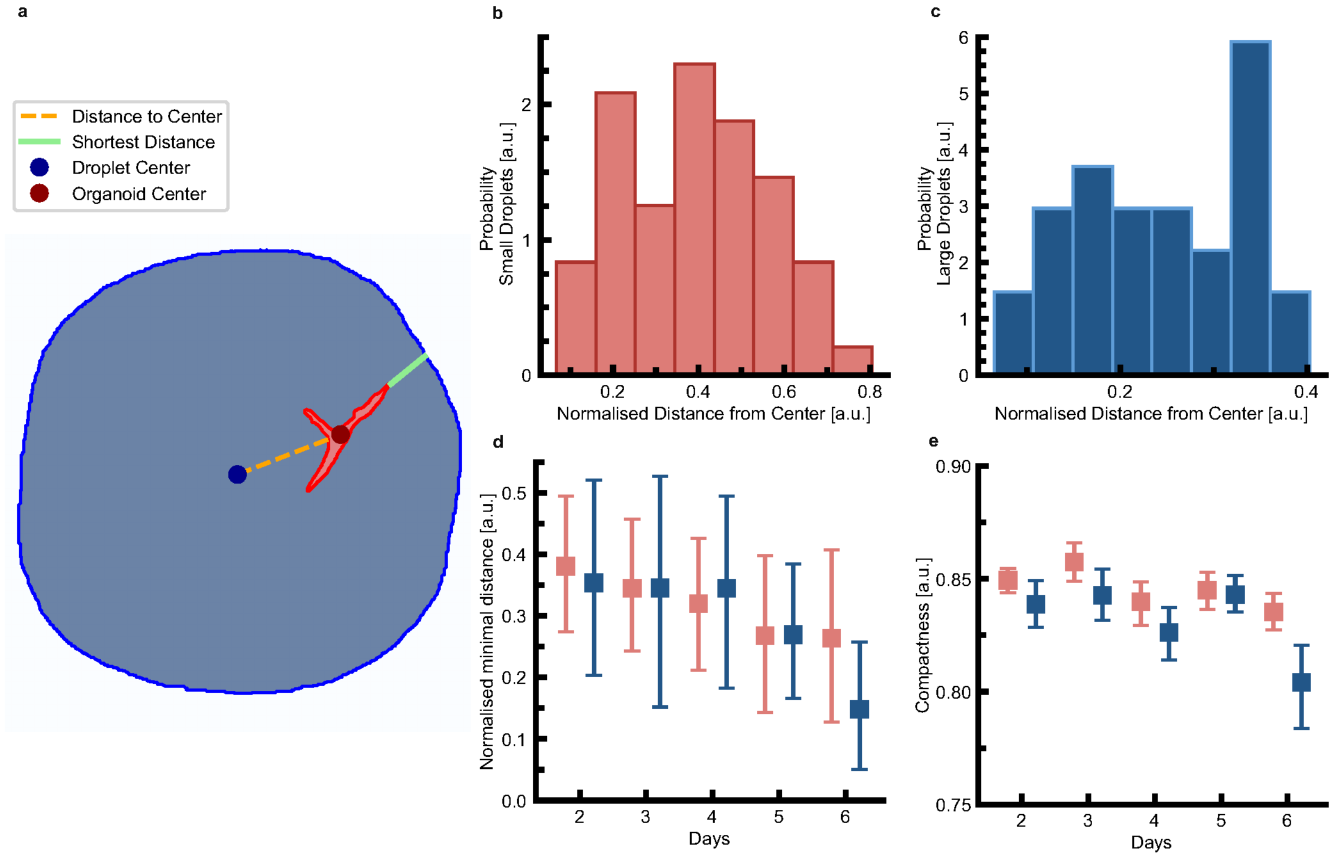

Figure S 5 Quantification of the spatial position of the organoid within the droplet. (a) The distance from the centre of the organoid to the centre of the collagen droplet is indicated by the orange dashed line. The green line shows the minimal distance from the periphery of the organoids to the outer edge of the droplet. (b) The normalised distance of the organoids to the centre of the small droplets on day 2 (n_{Small} = 52 organoid containing droplets were evaluated). (c) The normalised distance of the organoids to the centre of the large droplets on day 2 (n_{Large} = 32 organoid containing droplets were evaluated). (d) The normalised minimal distance over time for organoids grown in large and small droplets. The error bars show the 95% confidence interval ($n_{Large} \geq 14$ and $n_{Small} \geq 23$ for each day). (e) The compactness over time for large and small droplets. The error bars show the 95% confidence interval ($n_{Large} \geq 14$ and $n_{Small} \geq 23$ for each day).

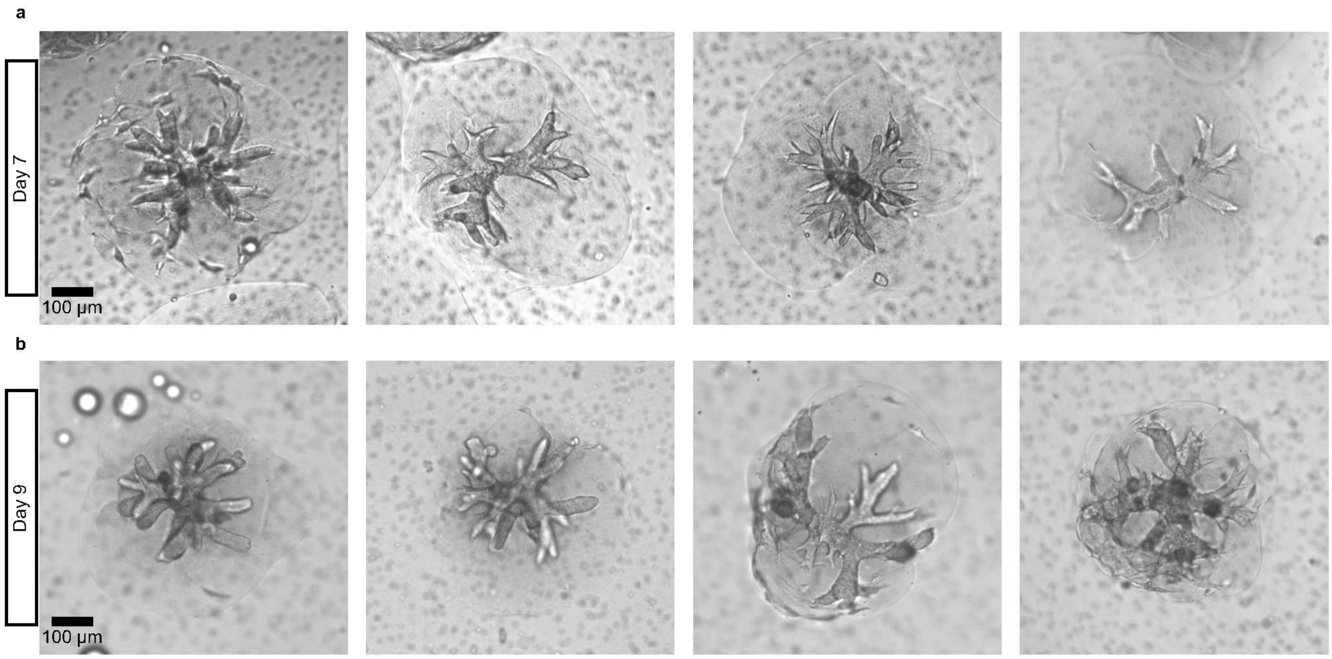

Figure S 6 Morphology of PDAC organoids cultured in large droplets beyond day 6. (a) Brightfield images of organoids grown in large collagen droplets on day 7 (n = 2 independent experiments). (b) Brightfield images of organoids grown in large collagen droplets on day 9 (n = 2 independent experiments).

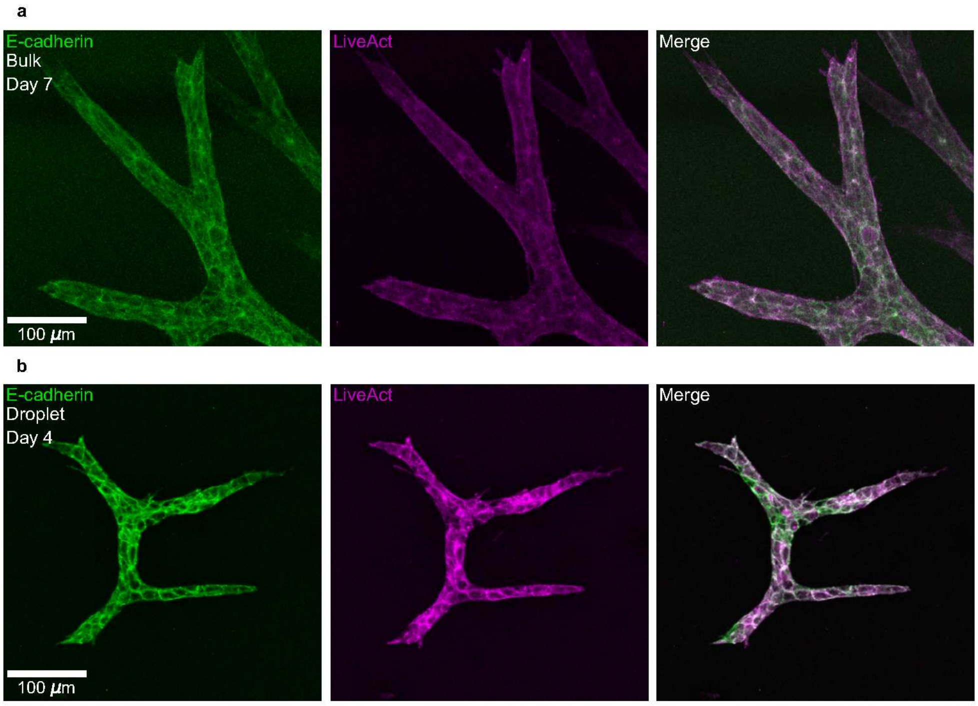

Figure S 7 Droplet-grown organoids on day 4 lack epithelialisation. (a) The bulk-grown organoid on day 7 displays a homogeneous distribution of E-Cadherin and F-actin throughout its branches. The cells in the branches display a mesenchymal phenotype in concordance with the organoid's developmental phase (n = 3 independent experiments). (b) The droplet-grown organoid on day 4 shows a homogeneous distribution of E-Cadherin and F-actin throughout the branches and displays a mesenchymal phenotype (n = 3 independent experiments).

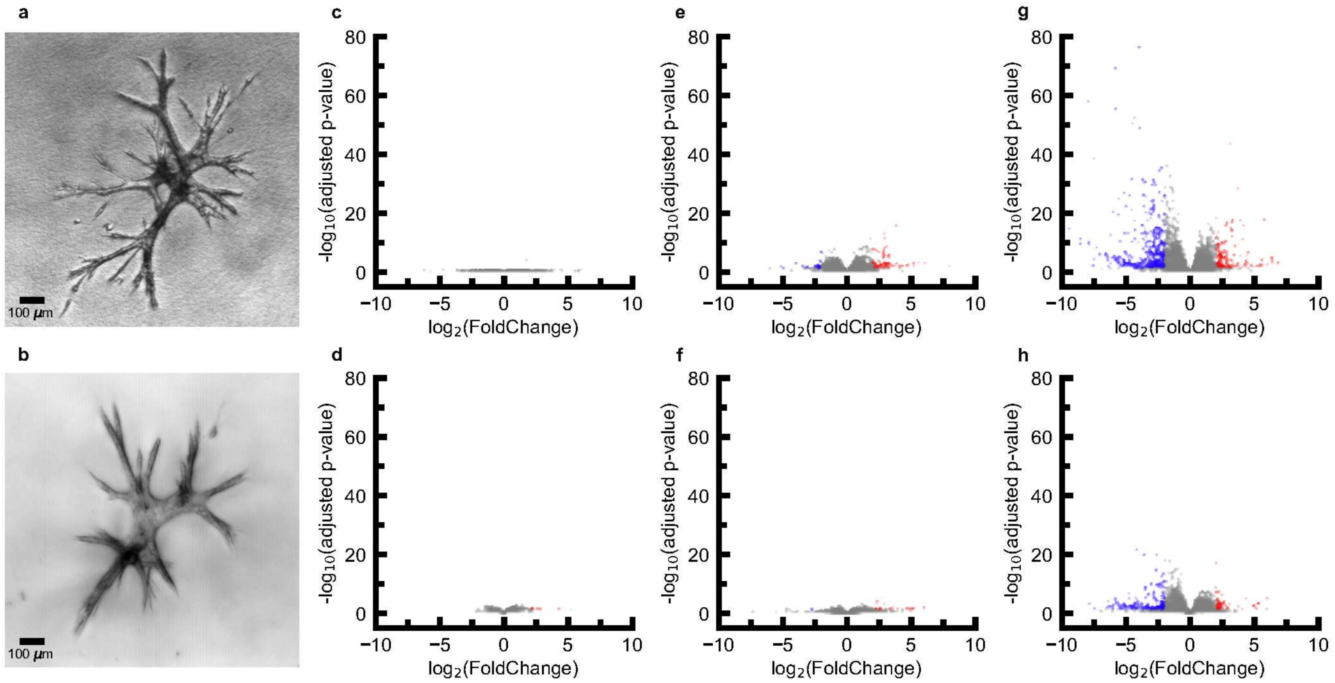

Figure S 8 Impact of collagen concentration and culture conditions on PDAC organoids. (a) Representative brightfield image of PDAC organoids cultured in 1.3 mg/ml bulk collagen gels on day 8 (n = 2 independent experiments). (b) Representative brightfield image of PDAC organoids cultured in 3 mg/ml bulk collagen gels on day 8 (n = 2 independent experiments). (c) Volcano plot showing significantly differentially expressed genes between organoid cultures in 3 mg/ml and 1.3 mg/ml collagen on day 4 (n = 4 independent experiments). (d) Volcano plot showing significantly differentially expressed genes between bulk organoid cultures in 3 mg/ml and 1.3 mg/ml collagen on day 7 (n = 4 independent experiments). (e) Volcano plot showing significantly differentially expressed genes between organoids grown in large and small droplets on day 4 (n = 4 independent experiments). (f) Volcano plot showing significantly differentially expressed genes between organoids grown in large and small droplets on day 7 (n = 4 independent experiments). (g) Volcano plot showing significantly differentially expressed genes between bulk organoid cultures in 1.3 mg/ml collagen and large droplet-based organoids in 3 mg/ml collagen on day 4 (n = 4 independent experiments). (h) Volcano plot showing significantly differentially expressed genes between bulk organoid cultures in 1.3 mg/ml collagen on day 7 and large droplet-based organoids in 3 mg/ml collagen on day 4 (n = 4 independent experiments).
